# Morphometric Characteristics of the Skull in Horses and Donkeys—A Pilot Study

**DOI:** 10.3390/ani10061002

**Published:** 2020-06-08

**Authors:** Katrina Merkies, Georgios Paraschou, Paul Damien McGreevy

**Affiliations:** 1 Department of Animal Biosciences, University of Guelph, Guelph, N1G 2W1 ON, Canada; 2 Campbell Centre for the Study of Animal Welfare, University of Guelph, Guelph, N1G 2W1 ON, Canada; 3 The Donkey Sanctuary, Sidmouth, Devon EX10 0NU, UK; georgios.paraschou@thedonkeysanctuary.org.uk; 4 Sydney School of Veterinary Science, Faculty of Science, University of Sydney, Sydney, NSW 2006, Australia; paul.mcgreevy@sydney.edu.au

**Keywords:** olfactory bulb, skull morphology, breed differences, hair whorl

## Abstract

**Simple Summary:**

Horses and donkeys are often lumped together but as separate species; they differ from each other in many ways. We examined the head characteristics of deceased horses and donkeys and measured various structures of both the skull and the brain. We calculated various ratios of skull measurements from horses and donkeys to be able to compare them to each other. We noted that donkey skulls have a larger forehead than horses. The olfactory bulb was smaller in donkeys and was rotated more forward than in horses. Most strikingly, we noted that the hair whorl on the forehead of horses almost always corresponded with the location of the olfactory bulbs, but in donkeys, the hair whorl was located much further down the nose. While it is unclear why these differences exist, it may relate to some of the striking differences in behaviour and physiology that have also been noted between donkeys and horses.

**Abstract:**

Horses and donkeys belong to the genus Equus, but important differences exist between the species, many of which affect their management and welfare. This study compared skull morphology between horses and donkeys. Horse (*n* = 14) and donkey (*n* = 16) heads were obtained post-mortem, sectioned sagittally close to the midline, and photographed for subsequent measurement of various skull structures. Skull, cranial, nasal, and profile indices were calculated for topographical comparisons between the species. The olfactory bulb area (OBA), OB pitch (the angle between the hard palate and the OB axis), and whorl location (WL) were also measured. A General Linear Model determined the main effect of species with Sidak’s multiple comparisons of species’ differences among the various measurements. There was no species difference in cranial or nasal indices (*p* > 0.13), but donkeys had a larger cranial profile than horses (*p* < 0.04). Donkeys had a smaller OBA (*p* < 0.05) and a steeper OB pitch (*p* < 0.02) than horses. The WL corresponded to the level of the OB in horses but was extremely rostral in donkeys (*p* < 0.0001). These results show clear differentiation in skull morphology between horses and donkeys. This may be useful in validating other physiological and behavioural differences between horses and donkeys.

## 1. Introduction

Since equids were domesticated, over 5000 years ago, humans have given them diverse roles ranging from work and transportation to sport and leisure. The selection of breeding animals has focused on specific traits such as colour, conformation, behaviour, and personality. Over time, such selection pressures have given rise to a wide variety of breeds with highly diverse physical characteristics from bulky draft horses to fine-boned Arabians, to miniature horses, to donkeys and mules.

Equine intraspecific diversity is evident in size, shape, colouration, conformation and, behavioural suitability for a particular purpose. Manifestations of selection pressures in various breeds have been documented in reports that focus on colour [1], genetics [2], anatomy [3], physiology [4], reproduction [5], trainability [6], personality [7], and behaviour [8]. Even within a breed, conformational differences are evident depending on the geographical region of origin [9,10].

Breed types can be broadly categorised according to their typical conformational characteristics based on genetic heritage. Cold-blooded horses refer to those from draft lineage, generally exhibiting a so-called coarse head, short neck, broad chest, and muscular body [9,11] compared to hot-blooded horses who descend mainly from Thoroughbred and Arabian lines. Thoroughbreds demonstrate longer withers, and Arabians have more sloping shoulders than draft horses [12]. Hot-blooded horses (e.g., Arabians) have a more concave head profile, while warm- to cold-blooded horses (e.g., Standardbreds) have relatively more convex profiles and longer skulls [13]. Warmbloods, originating from agricultural stock, changed their breeding goals over time to a lighter sport horse type, presenting a conformational mix between hot- and cold-bloods [14].

Breed types also show specific variations in behaviour and temperament. The Standardbred, as an example of a cold-blooded-type horse, is less reactive and quicker to habituate to aversive stimuli than more refined breeds, such as Thoroughbreds [7]. Cold-blooded horses are easier to handle [15] while Thoroughbreds are said to be more nervous [16], and take longer to learn a training task than other breeds [17]. Breeders of the Austrian Noriker draft horse have a firm belief in the effect of coat colour on temperament [9], for example, black Norikers are considered more lively.

Horses and donkeys belong to the same family of Equidae. However, within the genus Equus, there are clear variances in anatomical structure and behaviour of the various species. While donkeys and Standardbred racehorses both typically exhibit convex nasal profiles, the physiology and behaviour of these two equids is markedly different. Donkeys, evolving from arid climates, are more affected by intemperate weather than horses, but are less reactive to biting insects [18]. Donkeys can thrive on lower quality forage than horses, but this also puts them at risk for metabolic diseases when donkey owners feed them like horses [19,20,21]. While horses show more obvious behavioural responses to pain than donkeys [22,23], both donkeys and mules show more metabolic changes indicative of stress than horses when subjected to transportation and mixing at auction houses [24]. 

It has been suggested that behavioural differences among dog breeds may be predicted by skull size and shape [25,26,27]. Thus, phenotypic and behavioural dissimilarities among different horse breeds may also be reflected through skull morphology. The objective of this preliminary research is to determine differences in skull morphology between the Standardbred (as a cold-blooded representative) and donkeys, as a starting point for further investigation into the brain organisation that may underlie behavioural differences.

## 2. Materials and Methods 

### 2.1. Subjects and Head Preparation

No approval for the use of animals in research was obtained as the research dealt only with material from deceased subjects. All horses and donkeys used in this research were euthanized for reasons unrelated to the research project. Heads of mature Standardbred-type horses (*n* = 14) and mature donkeys (*n* = 16) were obtained post-mortem. Donkeys ranged in size from standard to so-called mammoth. Ages of the horses were estimated from an assessment of their dentition while ages of the donkeys were known. The estimated age of the horses ranged from 3–16 years (mean 7.5 ± 4.5 years). The age of the donkeys ranged from 3–38 years (mean 21 ± 10 years). The skulls were separated from the body by cutting through the proximal end of the first cervical vertebrae using a band-saw. Each skull was sectioned sagittally as close to the midline as possible, and each section was weighed. No other preparation was performed on the skulls. Coloured pins were inserted to mark topographical landmarks (occipital crest, olfactory bulb, orbital notch, incisive bone), and a ruler was placed along the hard palate to reference a horizontal dimension and provide a known length for measurement calibration. Photographs of the sectioned skulls were taken (Canon Powershot SD940 IS 12.1MP; Ottawa, ON, Canada) for retrospective measurement of various structures using the open-source software Image J (v1.46r; Madison, WI, USA).

### 2.2. Skull Measurements

**Figure 1:**
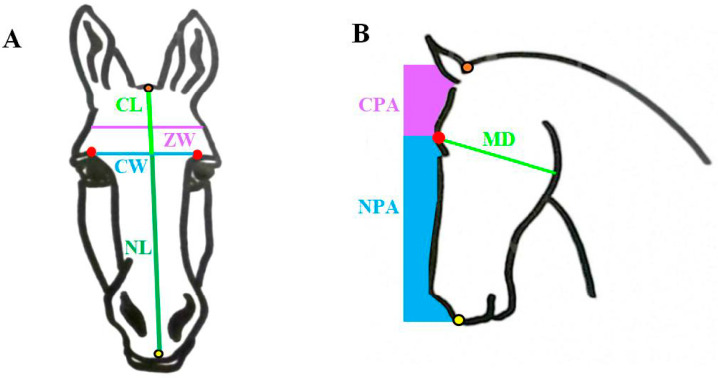
Diagram of the various measurements taken from horse (*n* = 14) and donkey (*n* = 16) skulls. The orbital notch is marked by the red dot. The occipital crest is marked by the orange dot, and the incisive bone is marked by the yellow dot. (**A**): The frontonasal suture line is estimated by a horizontal line joining the left and right orbital notches to determine cranial width = CW blue line. Cranial length = CL light green line; nasal length = NL dark green line; zygomatic width = ZW purple line. (**B**): mandibular depth = MD green line, nasal profile area = NPA blue area, cranial profile area = CPA purple area. See text for details of each measure.

**Figure 2:**
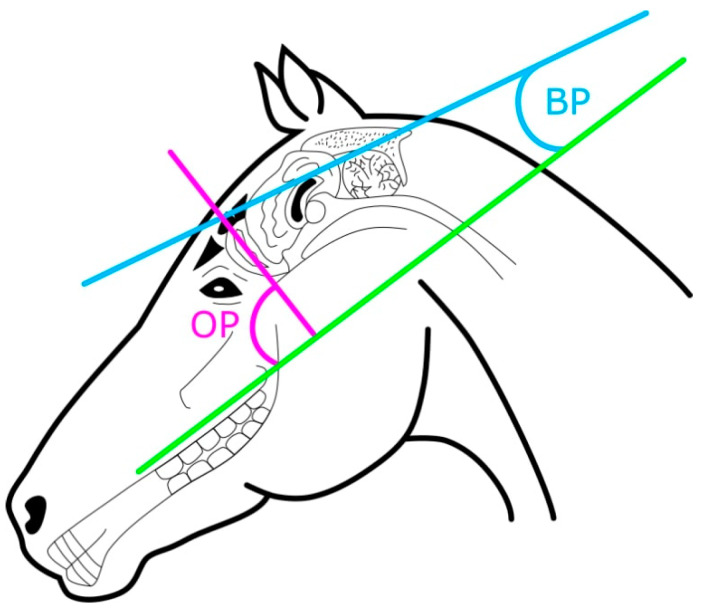
Diagram of brain measurements on horse (*n* = 14) and donkey (*n* = 16) skulls. The olfactory pitch (OP) was measured as the angle between the hard palate (green line) and the longitudinal axis of the olfactory bulb (purple line). The brain pitch (BP) was measured as the angle between the hard palate and the longitudinal axis of the brain (blue line).

Skull measurements included: cranial length (measured from the occipital crest to the frontonasal suture line), nasal length (measured from the rostral point of the incisive bone to the frontonasal suture line), skull length (sum of cranial length + nasal length), zygomatic width (sum of left and right sections measured from the zygomatic bone to the midline), cranial width (sum of left and right sections measured from the orbital notch to the midline along the frontonasal suture line) (Figure 1A), mandibular depth (measured from the orbital notch to the widest part of the mandible; Figure 1B), and whorl location (described by the facial distance between the rostral tip of the olfactory bulb (OB) and the centre of the forehead whorl). The frontonasal suture line was estimated by a line perpendicular to the orbital notch. Brain measurements included the OB pitch (the angle between the hard palate and the OB’s longitudinal axis), the brain pitch (the angle between the hard palate and the longitudinal axis of the cerebral hemispheres) (Figure 2), and the OB area estimated from the right olfactory bulb in situ. For skull length, nasal length, cranial length, mandibular depth, OB pitch, and brain pitch, averages were calculated from the measurements from the left and right skull sections. Raw data are available in Table S1.

From these measurements, the following indices were calculated as described by Evans and McGreevy [13], using the relevant averages from the above measurements:

skull index (SI) = zygomatic width × 100/skull length

cranial index (CI) = cranial width × 100/cranial length

nasal index (NI) = zygomatic width × 100/nasal length

mandibular index (MI) = mandibular depth × 100/skull length

nasal profile area (NPA) = rectangular area defined by 80 mm vertical line from the orbital notch to the rostral point of incisive bone (Figure 1B)

nasal profile index (NPI) = NPA/nasal length

cranial profile area = rectangular area defined by 80 mm vertical line from the orbital notch to the occipital crest (Figure 1B)

cranial profile index (CPI) = CPA/cranial length

For NPA and CPA, averages were calculated from the measurements from the left and right skull sections.

### 2.3. Statistical Analyses 

All brain measures and calculations were analysed using SAS (v9.4, SAS Institute, Cary, NC, USA). Pearson correlations determined any relationships between skull morphology and brain measurements. A General Linear Model determined the main effect of species with Sidak’s multiple comparisons of species differences among the various measurements. 

## 3. Results

### 3.1. Horses

The results of the morphometric measurements of the horse skulls are presented in Table 1. There was large variability in the measurements among individual horses. For example, NI had a mean of 76.1 ± 14.4, with measurements ranging from 63.6 to 108.3. The brain pitch and olfactory pitch also exhibited a wide range (2.3° to 176.5° and 51.4°to 75.9°, respectively). The olfactory bulb pitch showed a trend of being correlated to cranial width (Χ^2^ (1, *n* = 12) = −0.55, *p* = 0.063). The location of the whorl aligned to the location of the olfactory bulbs within 1.5 cm. The whorl location tended to be correlated weakly to nasal length (Χ^2^ (1, *n* = 14) = −0.49, *p* = 0.078) and brain pitch (Χ^2^ (1, *n* = 14) = 0.48, *p* = 0.08).

### 3.2. Donkeys

The results of the morphometric measurements of the donkey skulls are presented in Table 1. A greater range in measurements was seen in the donkeys as the animals in the current sample ranged in size from standard to so-called mammoth. In particular, skull length and cranial length varied widely (40.3–54.9 cm and 16.3–24.9 cm, respectively). The location of the whorl was almost exclusively located quite rostrally. The whorl location was correlated to NPI (Χ^2^ (1, *n* = 12) = 0.66, *p* < 0.019), skull length (Χ^2^ (1, *n* = 14) = 0.75, *p* < 0.002) and nasal length (Χ^2^ (1, *n* = 14) = 0.74, *p* = 0.0023).

### 3.3. Comparison of Measurements between Donkeys and Horses

Table 1 shows the statistical outcomes of the skull morphometrics between species. Donkeys had smaller heads than horses, as reflected in lower skull weights (F (1.18) = 26.38, *p* < 0.0001), a smaller cranial width (F (1.15) = 42.67, *p* < 0.0001), and mandibular depth (F (1.21) = 13.05, *p* = 0.0019). Donkeys had shorter heads than horses, mostly due to a shorter cranial length (F (1.23) = 51.49, *p* = 0.0002), given that there was no statistical difference in their nasal lengths (F (1.23) = 2.09, *p* > 0.19). The calculated indices provide a better comparison between the species because donkeys ranged in size much more than the horses did. There was no species difference in cranial index, nasal index, or mandibular index (*p* > 0.13), but donkeys had a larger skull index than horses (F (1.15) = 14.01, *p* = 0.0025). Donkeys had a smaller CPI than horses (F (1.21) = 7.54, *p* < 0.034), but there was no difference in NPI (F (1.21) = 0.05, *p* > 0.83). Donkeys also had a smaller OB area (F (1.13) = 4.96, *p* < 0.05) and a smaller OB pitch (F (1.15) = 7.60, *p* = 0.0163) than horses, although there was no difference in the brain pitch (F (1.23) = 0.69, *p* > 0.43). The greatest difference was seen in the location of the whorl, which corresponded to the level of the OB bulb in horses, but was located extremely rostrally in donkeys (F (1.21) = 56.28, *p* < 0.0001). Age (*p* > 0.08) and sex (*p* > 0.09) were not statistically significant for any of the measures.

## 4. Discussion

These preliminary results show that donkeys and Standardbred horses have similarly shaped heads, although donkeys have smaller heads and a more distinct forehead than horses. Donkeys also have smaller olfactory bulb areas than horses. While the orientation of the brain does not significantly differ between horses and donkeys, the olfactory bulbs in donkeys are rotated more rostrally. Facial whorls in donkeys are located lower down the face while in the current series of horses, they are in close proximity to the olfactory bulb. Anatomical differences between horses and donkeys have been noted in the jaws and teeth [19], but this is the first report of morphometric differences in skull morphology.

**Figure 3:**
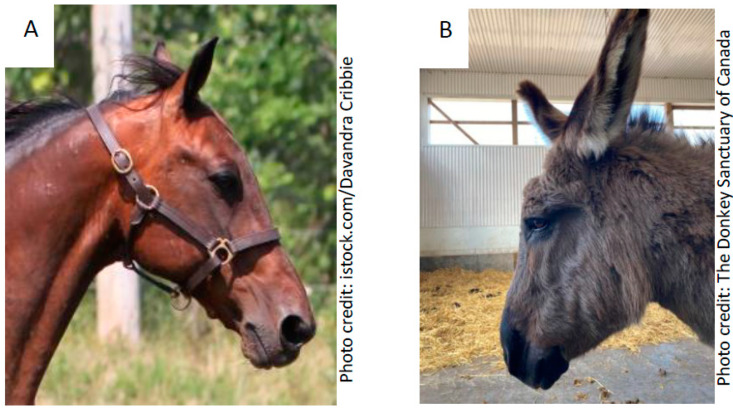
Comparison between the Standardbred (**A**) and donkey (**B**) head profiles. Donkeys have shorter and narrower faces than Standardbreds, but they did not differ in the nasal, cranial or, mandibular indices. While the nasal profile index also did not differ, donkeys did exhibit a smaller cranial profile index, as portrayed by the distinct forehead in the donkey.

Values obtained for horse skull measurements in the current study corresponded quite closely to those previously obtained for Arabians [28], and Standardbreds [13]. However, Evans and McGreevy [13] demonstrated that Standardbreds had longer and wider skulls than Arabians or Thoroughbreds. This difference might be attributed to differing measurements since Cervantes et al. [28] did not indicate landmarks from which they conducted their head length and width measurements. The relatively large head of the Standardbred may have accentuated the differences in skull length and width between the Standardbred horses and donkeys measured in the current study. The use of indices helped to account for these variations in absolute size, and given that there was no difference between Standardbred horses and donkeys in the nasal, cranial, or mandibular indices, it appears that donkeys and Standardbred horses have similar ratio aspects. However, the skull index did differ, showing donkeys to have somewhat shorter and narrower faces than Standardbred horses. In contrast, the nasal profile index did not differ between Standardbred horses and donkeys, indicating they both exhibit a more convex profile. That said, donkeys did exhibit more convexity than Standardbred horses in the cranial profile, as is often manifested by their distinct forehead (Figure 3). Interestingly, while others have indicated a greater variability in measurements of horse skulls due to the nasal component [9,13,29,30], here we show greater variability in the cranial component between Standardbred horses and donkeys. 

Selective breeding in dogs has similarly resulted in great diversity in conformation across different breeds, notably seen in the range of facial features. Skull length can range from 7–28 cm [31,32]. Brachycephaly has resulted not only in a frontal rotation of the brain of domestic dogs [32] but in shifts of typical behaviour. For dog owners, this implies higher levels of fear and increased chasing behaviour in long-nosed dogs suitable for hunting, while short-nosed dogs appear to have the capacity for increased visual focus on human cues suitable for companion animals [26,27]. These behavioural differences may be the result of accompanying differences in both brain organization and retinal structure. In dogs, the concentration of retinal ganglion cells is positively correlated to the skull shape and the eye size, with a more concentrated distribution in the equivalent of the area centralis in short-nosed dogs, possibly indicating increased visual acuity in the centre of the visual field [31]. In contrast, long-skulled dogs have a visual streak with ganglion cells distributed across the retina in a band that suggests good peripheral vision. Correspondingly, horse breeds with longer heads display increased retinal ganglion cell density along with their equivalent of a visual streak [33]. 

Standardbred horses are predominantly bay, which may even be a contributing factor to their skull morphometrics. While the Austrian Noriker draft horse has larger absolute skull measurements than Standardbred horses due to its larger overall size, researchers have demonstrated differences in head size in that breed that are dependent on colour; bay or black horses have longer noses than tobiano Noriker horses, and roans have smaller heads [9].

The current study provides the first reported finding of an external landmark for the position of the olfactory bulbs, which are always found in close proximity to the facial whorl in horses, while in donkeys, the whorl is located extremely rostral to the olfactory bulbs. Despite having a relatively larger cranial space, donkeys; nevertheless, have smaller olfactory bulbs than Standardbred horses. Olfaction plays a key role in social interactions, such that in rodents, the removal of olfactory cues through cage cleaning causes an increase in anxiety and aggression [34]. Olfaction is the second-most represented behaviour between jennies and their new-born foals (only behind visual observation) [35], resulting in changes in the neural structure of the olfactory bulb affecting memory that drives maternal recognition and bonding critical to survival [34]. In honey bees, the olfactory bulb is responsible for identifying odours that stimulate memory to help relocate food sources [36].

Olfactory stimuli are processed in the amygdala, which is also a centre for processing emotional stimuli [37]. The amygdala, among other roles, regulates feeding behaviour through odour intensity, enhances memory performance through emotions elicited from odours, particularly in response to aversive stimuli [38], and is important in associative learning [39,40]. The hippocampus also responds to olfactory stimuli and plays a role in long-term memory, emotional experience, and response to stress [38]. For example, mice show higher activity in the dorsal hippocampus when detecting familiar odours in a novel environment, but this ability is impaired with olfactory lesions [41]. The significance of the olfactory bulb size and angle to species differences is unclear. However, horses have significantly larger olfactory bulbs than humans and are able to detect a wider range of odours [42]. Further studies in equids on the impact of olfactory memory on behavioural responses in relation to the location of their olfactory bulbs may prove interesting. Given that population laterality in nostril use has been reported in horses [43], it may pay to explore the evidence for a similar attribute in donkeys.

It appears that in Standardbred horses, at least, the location of the olfactory bulb can be inferred by the forehead whorls. This may have relevance to documented temperament differences based on whorl location. In cattle, whorls located above the eye line (that can be drawn between the medial canthi) are related to individuals who are more reactive when placed in a squeeze chute [44]. In horses, forehead hair whorls have been used to predict the temperament of a horse since ancient times [45]. Anecdotally, horses with whorls located below the eye level indicate so-called intelligence, while whorls located in the centre of the forehead present a so-called uncomplicated personality [45]. While whorls can vary greatly in size and shape, the location of the forehead whorl is a highly heritable trait (h = 0.83 [46]), and horses with whorls located above the eye line are harder to manage [47]. Associations between the development of neural tissue and hair whorls have been suggested as the processes occur simultaneously during embryonic development [48]. Furthermore, the direction of hair in the whorl pattern correlates with laterality, with clockwise whorls predicting left side bias and counter clockwise whorls predicting right-side bias in horses presented with a novel object [48]. To the authors’ knowledge, there are no published studies of any relationship between whorl location and temperament in donkeys, but the notion that lower whorls are related to calmer temperaments may align with anecdotal observations that donkeys, in general, are less reactive than horses. 

Temperament in horses is a very important trait related to a horse’s usefulness for its selected purpose [49]. Researchers have demonstrated specific temperament traits related to dog-specific breeds [50,51], so it follows that specific horse breeds would also tend to display certain behavioural traits. Temperament has been investigated in a number of horse breeds, and researchers have demonstrated a more reactive temperament in Thoroughbreds and Arabians than in cold-blooded horses [52,53]. Thoroughbreds take longer to perform a stepping backward test than other breeds [17], indicating some relationship between breed and cognition. The hot-blooded ancestry of Thoroughbreds and Arabians contributes to higher levels of anxiousness, excitability, sociability, and inquisitiveness compared to Quarter Horses, Appaloosas, and Irish Draft Horses [7,16]. These breeds also exhibit particular conformation as reinforced by stud books and breed standards, thus conformation and temperament are quite likely genetically linked [54]. 

Temperament in donkeys differs from horses in that they are generally less overtly reactive, leading them to be defined as stoic [23]. Donkeys generally live alone or in very small groups, thus do not exhibit typical “herd” behaviour like horses [19]. They may be territorial and display aggression toward other species sharing their space [19]. While generally regarded as a flight animal, donkeys’ resort to fight behaviour much more readily than horses. Donkeys may also assume a “freeze” posture when presented with frightening stimuli, which may be defined as “stubborn” by some [19]. However, although donkeys may take more time, they are more adept at solving puzzles than horses [55]. Similarly, Baragli and Regolin [56] showed donkeys to be proficient at finding a hidden object while Standardbred horses in the same study failed to do so. Anatomically, donkeys differ from horses with their large ears, short neck, and small feet [19], and coupled with differences in skull morphometrics shown in the current study; this may explain some of the behavioural differences between the two species. However, it is important to bear in mind that environment and individual experience have a significant impact on how an animal responds to any particular stimuli.

## 5. Conclusions

This pilot study compared the skull morphology of horses and donkeys, revealing that donkeys have a more distinct forehead, and yet have smaller olfactory bulbs rotated more rostrally compared to horses. Further, the facial whorl in donkeys is not associated with the location of the olfactory bulbs as it is in horses. These results may be linked to other documented differences between horses and donkeys, particularly behavioural differences. Future research exploring possible links between skull morphology and behaviour may result in better prediction of behaviours based on more than merely breed or species. Thus, horses and donkeys of a given morphotype could be better matched to a job or discipline suitable to their temperament, improving their welfare, increasing the safety of humans working with them, and reducing behavioural wastage due to miscommunication with humans. 

## Figures and Tables

**Table 1:** Morphometric measurements (mean ± SD) of horse (*n* = 12) and donkey (*n* = 14) skulls. *P* values were calculated using a General Linear Model, with a value <0.05 considered significantly different.

Variable	Horse	Donkey	*p*-Value
	Mean	Mean	
Head weight (kg)	22.5 ± 4.9	13.6 ± 3.7	<0.0001 *
Skull length (cm)	53.6 ± 2.6	46.6 ± 5.0	0.0080 *
Cranial length (cm)	23.6 ± 1.4	20.4 ± 2.7	0.0002 *
Nasal length (cm)	30.4 ± 3.9	27.5 ± 3.1	0.1919
Zygomatic width (cm)	22.6 ± 1.8	21.9 ± 2.2	0.0776
Cranial width (cm)	19.0 ± 0.8	16.3 ± 2.4	<0.0001 *
Mandibular depth (cm)	27.2 ± 1.1	24.5 ± 2.4	0.0019 *
Skull index	42.2 ± 3.2	46.2 ± 3.4	0.0025 *
Cranial index	80.9 ± 6.2	77.2 ± 9.1	0.1399
Nasal index	76.0 ± 14.5	79.6 ± 7.5	0.6600
Mandibular index	50.8 ± 2.6	52.2 ± 5.2	0.5791
Cranial profile index	8.8 ± 1.0	7.6 ± 1.2	0.0335 *
Nasal profile index	9.1 ± 2.8	7.3 ± 0.8	0.8359
Olfactory bulb area (cm^2^)	2.3 ± 1.3	1.98 ± 1.3	0.0478 *
Olfactory bulb pitch (°)	66.0 ± 11.8	56.6 ± 1318	0.0163 *
Brain pitch (°)	18.3 ± 45.7	16.7 ± 37.8	0.4338
Whorl location (cm)	0.1 ± 1.5	8.0 ± 6.5	0.0001 *

* denotes the statistical difference between horses and donkeys at the level of *p* < 0.05.

## Supplementary Materials

The following are available online at https://www.mdpi.com/2076-2615/10/6/1002/s1, Table S1: Raw morphometric data from horse and donkey skulls.

## References

[1] Grilz-Seger G., Reiter S., Neuditschko M., Wallner B., Rieder S., Leeb T., Jagannathan V., Mesarič M., Cotman M., Pausch H. (2020). A genome-wide association analysis in noriker horses identifies a snp associated with roan coat color. J. Equine Vet. Sci..

[2] Aberle K.S., Hamann H., Drögemüller C., Distl O. (2004). Genetic diversity in german draught horse breeds compared with a group of primitive, riding and wild horses by means of microsatellite dna markers. Anim. Genet..

[3] Robert C., Valette J.-P., Denoix J.-M. (2013). Longitudinal development of equine forelimb conformation from birth to weaning in three different horse breeds. Vet. J..

[4] Malinowski K., Christensen R.A., Hafs H.D., Scanes C.G. (1996). Age and breed differences in thyroid hormones, insulin-like growth factor (igf)-i and igf binding proteins in female horses. J. Anim. Sci..

[5] Gastal E.L., Gastal M.O., Beg M.A., Neves A.P., Petrucci B.P.L., Mattos R.C., Ginther O.J. (2008). Miniature ponies: Similarities and differences from larger breeds in follicles and hormones during the estrous cycle. J. Equine Vet. Sci..

[6] Janczarek I., Stachurska A., Kedzierski W., Wilk I. (2013). Responses of horses of various breeds to a sympathetic training method. J. Equine Vet. Sci..

[7] Lloyd A.S., Martin J.E., Bornett-Gauci H.L.I., Wilkinson R.G. (2008). Horse personality: Variation between breeds. Appl. Anim. Behav. Sci..

[8] McGreevy P.D., Thomson P.C. (2006). Differences in motor laterality between breeds of performance horse. Appl. Anim. Behav. Sci..

[9] Druml T., Baumung R., Sölkner J. (2008). Morphological analysis and effect of selection for conformation in the noriker draught horse population. Livest. Sci..

[10] Goodwin D., Levine M., McGreevy P.D. (2008). Preliminary investigation of morphological differences between ten breeds of horses suggests selection for paedomorphosis. J. Appl. Anim. Welf. Sci..

[11] Folla F., Sartori C., Guzzo N., Pigozzi G., Mantovani R. (2019). Genetics of linear type traits scored on young foals belonging to the italian heavy draught horse breed. Livest. Sci..

[12] Merkies K., Alebrand J., Harwood B., LaBarge K., Scott L. (2020). Investigation into thoracic asymmetry in ridden horses. Comp. Exerc. Physiol..

[13] Evans K.E., McGreevy P.D. (2006). Conformation of the equine skull: A morphometric study. J. Vet. Med. Ser. C Anat. Histol. Embryol..

[14] Nolte W., Thaller G., Kuehn C. (2019). Selection signatures in four german warmblood horse breeds: Tracing breeding history in the modern sport horse. PLoS ONE.

[15] Górecka-Bruzda A., Jastrzebska E., Sosnowska Z., Jaworski Z., Jezierski T., Chruszczewski M.H. (2011). Reactivity to humans and fearfulness tests: Field validation in polish cold blood horses. Appl. Anim. Behav. Sci..

[16] Sackman J.E., Houpt K.A. (2019). Equine personality: Association with breed, use, and husbandry factors. J. Equine Vet. Sci..

[17] Fenner K., Freire R., McLean A., McGreevy P. (2019). Behavioral, Demographic, and Management influences on equine responses to negative reinforcement. J. Vet. Behav..

[18] Proops L., Osthaus B., Bell N., Long S., Hayday K., Burden F. (2019). Shelter-seeking behavior of donkeys and horses in a temperate climate. J. Vet. Behav..

[19] Burden F., Thiemann A. (2015). Donkeys are different. J. Eq. Vet. Sci..

[20] Davis E. (2019). Donkey and mule welfare. Vet. Clin. N. Am.-Eq. Pract..

[21] McLean A.K., Navas González F.J., Canisso I.F. (2019). Donkey and mule behavior. Vet. Clin. N. Am.-Eq. Pract..

[22] Grint N.J., Beths T., Yvorchuk-St Jean K., Whay H.R., Murrell J.C. (2017). Analysis of behaviors observed during mechanical nociceptive threshold testing in donkeys and horses. J. Equine Vet. Sci..

[23] van Dierendonck M.C., van Loon J.P.A.M., Burden F.A., Rickards K. (2020). Monitoring acute pain in donkeys with the equine utrecht university scale for donkeys composite pain assessment (equus-donkey-compass) and the equine utrecht university scale for donkey facial assessment of pain (Equus-Donkey-Fap). Animals.

[24] Corrales-Hernández A., Mota-Rojas D., Guerrero-Legarreta I., Roldan-Santiago P., Rodríguez-Salinas S., Yáñez-Pizaña A., de la Cruz L., González-Lozano M., Mora-Medina P. (2018). Physiological responses in horses, donkeys and mules sold at livestock markets. Int. J. Vet. Sci. Med..

[25] Georgevsky D., Carrasco J.J., Valenzuela M., McGreevy P.D. (2013). Domestic dog skull diversity across breeds, breed groupings, and genetic clusters. J. Vet. Behav..

[26] McGreevy P.D., Georgevsky D., Carrasco J., Valenzuela M., Duffy D.L., Serpell J.A. (2013). Dog behavior co-varies with height, bodyweight and skull shape. PLoS ONE.

[27] Stone H.R., McGreevy P.D., Starling M.J., Forkman B. (2016). Associations between domestic-dog morphology and behaviour scores in the dog mentality assessment. PLoS ONE.

[28] Cervantes I., Baumung R., Molina A., Druml T., Gutiérrez J.P., Sölkner J., Valera M. (2009). Size and shape analysis of morphofunctional traits in the spanish arab horse. Livest. Sci..

[29] Radinsky L. (1984). Ontogeny and phylogeny in horse skull. Evolution.

[30] Komosa M., Molinski K., Godynicki S. (2006). The variability of cranial morphology in modern horses. Zoolog. Sci..

[31] McGreevy P., Grassi T.D., Harman A.M. (2004). A strong correlation exists between the distribution of retinal ganglion cells and nose length in the dog. Brain. Behav. Evol..

[32] Roberts T., McGreevy P., Valenzuela M. (2010). Human induced rotation and reorganization of the brain of domestic dogs. PLoS ONE.

[33] Evans K.E., McGreevy P.D. (2007). The distribution of ganglion cells in the equine retina and its relationship to skull morphology. J. Vet. Med. Ser. C Anat. Histol. Embryol..

[34] Nielsen B.L. (2018). Making Sense of It All: The importance of taking into account the sensory abilities of animals in their housing and management. Appl. Anim. Behav. Sci..

[35] Mazzatenta A., Veronesi M.C., Vignola G., Ponzio P., Carluccio A., De Amicis I. (2019). Behavior of martina franca donkey breed jenny-and-foal dyad in the neonatal period. J. Vet. Behav..

[36] Simcock N.K., Gray H., Bouchebti S., Wright G.A. (2018). Appetitive olfactory learning and memory in the honeybee depend on sugar reward identity. J. Insect Physiol..

[37] Patin A., Pause B.M. (2015). Human amygdala activations during nasal chemoreception. Neuropsychologia.

[38] Soudry Y., Lemogne C., Malinvaud D., Consoli S.M., Bonfils P. (2011). Olfactory system and emotion: Common substrates. Eur. Ann. Otorhinolaryngol. Head Neck Dis..

[39] Cardinal R.N., Parkinson J.A., Hall J., Everitt B.J. (2002). Emotion and motivation: The role of the amygdala, ventral striatum, and prefrontal cortex. Neurosci. Biobehav. Rev..

[40] Corbit L.H., Leung B.K., Balleine B.W. (2013). The role of the amygdala-striatal pathway in the acquisition and performance of goal-directed instrumental actions. J. Neurosci..

[41] Pena R.R., Medeiros D. (2017). de C.; Guarnieri, L. de O.; Guerra, J.B.; Carvalho, V.R.; Mendes, E.M.A.M.; Pereira, G.S.; Moraes, M.F.D. Home-Cage odors spatial cues elicit theta phase/gamma amplitude coupling between olfactory bulb and dorsal hippocampus. Neuroscience.

[42] Saslow C.A. (2002). Understanding the perceptual world of horses. Appl. Anim. Behav. Sci..

[43] McGreevy P.D., Rogers L. (2005). Motor and sensory laterality in thoroughbred horses. Appl. Anim. Behav. Sci..

[44] Grandin T., Deesing M.J., Struthers J.J., Swinker A.M. (1995). Cattle with hair whorl patterns above the eyes are more behaviorally agitated during restraint. Appl. Anim. Behav. Sci..

[45] Meyer J.F. (2008). What’s in a whorl (hair swirls of horses). Horse Rider.

[46] Górecka A., Słoniewski K., Golonka M., Jaworski Z., Jezierski T. (2006). Heritability of hair whorl position on the forehead in konik horses. J. Anim. Breed. Genet..

[47] Górecka A., Golonka M., Chruszczewski M., Jezierski T. (2007). A note on behaviour and heart rate in horses differing in facial hair whorl. Appl. Anim. Behav. Sci..

[48] Shivley C., Grandin T., Deesing M. (2016). Behavioral laterality and facial hair whorls in horses. J. Equine Vet. Sci..

[49] Graf P., König von Borstel U., Gauly M. (2013). Importance of personality traits in horses to breeders and riders. J. Vet. Behav..

[50] Starling M.J., Branson N., Thomson P.C., McGreevy P.D. (2013). “Boldness” in the domestic dog differs among breeds and breed groups. Behav. Process..

[51] Mehrkam L.R., Wynne C.D.L. (2014). Behavioral Differences among breeds of domestic dogs (Canis Lupus Familiaris): Current status of the science. Appl. Anim. Behav. Sci..

[52] Janiszewska J., Ignor J., Cieśla A. (2004). Einfluss eines 11-monatigen trainings auf die ergebnisse des “Ängstlichkeitstests” von jungen halbblut-hengsten. Arch. Anim. Breed..

[53] Borstel U., Pirsich W., Gauly M., Bruns E. (2012). Repeatability and reliability of scores from ridden temperament tests conducted during performance tests. Appl. Anim. Behav. Sci..

[54] Kuhnke S., Bär K., Bosch P., Rensing M., Borstel U.K.V. (2019). Evaluation of a novel system for linear conformation, gait, and personality trait scoring and automatic ranking of horses at breed shows: A pilot study in american quarter horses. J. Equine Vet. Sci..

[55] Osthaus B., Proops L., Hocking I., Burden F. (2013). Spatial cognition and perseveration by horses, donkeys and mules in a simple a-not-b detour task. Anim. Cogn..

[56] Baragli P., Regolin L. Cognition tests in equids (Equus Caballus and Equus Asinus). Proceedings of the International Equine Science Meeting.

